# Freestanding Nanolayers of a Wide‐Gap Topological Insulator through Liquid‐Phase Exfoliation

**DOI:** 10.1002/chem.202004320

**Published:** 2020-12-04

**Authors:** Mai Lê Anh, Pavel Potapov, Daniel Wolf, Axel Lubk, Bernhard Glatz, Andreas Fery, Thomas Doert, Michael Ruck

**Affiliations:** ^1^ Faculty of Chemistry and Food Chemistry Technische Universität Dresden 01062 Dresden Germany; ^2^ Leibniz IFW Dresden 01069 Dresden Germany; ^3^ Würzburg-Dresden Cluster of Excellence ct.qmat Technische Universität Dresden 01062 Dresden Germany; ^4^ Leibniz Institute of Polymer Research Dresden 01069 Dresden Germany; ^5^ Max Planck Institute for Chemical Physics of Solids 01187 Dresden Germany

**Keywords:** 2D materials, delamination, exfoliation, topochemistry, topological insulators

## Abstract

The layered salt Bi_14_Rh_3_I_9_ is a weak three‐dimensional (3D) topological insulator (TI), that is, a stack of two‐dimensional (2D) TIs. It has a wide non‐trivial band gap of 210 meV, which is generated by strong spin‐orbit coupling, and possesses protected electronic edge‐states. In the structure, charged layers of ${}_{\infty }{}^{2}[$ (Bi_4_Rh)_3_I]^2+^ honeycombs and ${}_{\infty }{}^{1}[$ Bi_2_I_8_]^2−^ chains alternate. The non‐trivial topology of Bi_14_Rh_3_I_9_ is an inherent property of the 2D intermetallic fragment. Here, the exfoliation of Bi_14_Rh_3_I_9_ was performed using two different chemical approaches: (a) through a reaction with *n*‐butyllithium and poly(vinylpyrrolidone), (b) through a reaction with betaine in dimethylformamide at 55 °C. The former yielded few‐layer sheets of the new compound Bi_12_Rh_3_I, while the latter led to crystalline sheets of Bi_14_Rh_3_I_9_ with a thickness down to 5 nm and edge‐lengths up to several ten microns. X‐ray diffraction and electron microscopy proved that the structure of Bi_14_Rh_3_I_9_ remained intact. Thus, it was assumed that the particles are still TIs. Dispersions of these flakes now allow for next steps towards the envisioned applications in nanoelectronics, such as the study of quantum coherence in deposited films, the combination with superconducting particles or films for the generation of Majorana fermions, or studies on their behavior under the influence of magnetic or electric fields or in contact with various materials occurring in devices. The method presented generally allows to exfoliate layers with high specific charges and thus the use of layered starting materials beyond van der Waals crystals.

## Graphical abstract

**Flaky insulation**: Topological insulators are a novel class of quantum materials that have high application potential in quantum computers and spintronics. This work presents a scalable chemical protocol to exfoliate crystals of the topological insulator Bi_14_Rh_3_I_9_ into unsupported few layer thick flakes without decomposition. The new method is generally suitable to overcome strong electrostatic interactions in layered salts.

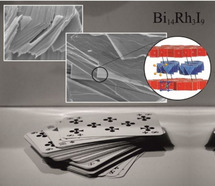

## Introduction

Two‐dimensional (2D) materials have attracted significant attention due to their physical properties that distinctly differ from bulk materials.[^1^, ^2^] In this context, graphene must be mentioned as the most famous 2D material with its outstanding properties such as high charge carrier mobility[^1^, ^3^] and thermal conductivity.^[4]^ While these properties suggest graphene as high‐end material for electronics and other applications, for example, in biotechnology,^[5]^ insufficient options to tune its band gap[^6^, ^7^, ^8^] and to control structural defects[^9^, ^10^] are limiting industrial applications. Inspired by the success of graphene, an enormous interest in the research of further 2D materials has been triggered. Thereby, many groups have concentrated on top‐down syntheses, facing the key challenge to control the structure–property relation on an atomic level. Using all the knowledge gained from the bulk material graphite, including the delamination strategies for graphene, other layered materials with weak van der Waals interactions have been successfully exfoliated. These include, for instance, *h*‐BN, which is isoelectronic to graphene,^[11]^ transition metal dichalcogenides like MoS_2_,^[12]^ and metal halides like PbI_2_.^[13]^ Beside, a different type of bulk material has been effectively delaminated via (harsh) wet‐chemical methods, the so‐called MAX phases.^[14]^ The most prominent compound is Ti_3_AlC_2_. These high‐melting metal carbides or nitrides have layered structures, but no van der Waals gaps. However, by etching with hydrofluoric acid the *A* component, in this case Al, can be removed topochemically and OH^−^ or F^−^ terminate the remaining transition metal carbide layers. In this way van der Waals gaps are generated, which allow the subsequent delamination of free‐standing metallic layers, named MXenes.^[15]^

Such 2D metals are of special interest, since quantum confinement leads to a density of state that markedly differs from bulk metals. As a consequence, the electronic resistance of 2D metals is independent of the path length. In contrast to the variety of semiconducting 2D materials, freestanding layers of metallic conductors are rare. The major reason for this is the scarcity of metallic bulk precursors with coherent networks of strong covalent bonds in only two dimension that are, for instance, separated by weak van der Waals gaps. In addition, monolayers of metal atoms are mostly only stable on substrate surfaces or with surfactants^[16]^ and typically show strong interactions with these components. Using carbon monoxide as a surface confining agent, the synthesis of freestanding Pd nanosheets succeeded that are about 10 atomic layers thick.^[17]^ Stacks of more or less free‐standing metallic nanosheets of Ag were obtained by repeated folding and calendering (RFC) of two side‐by‐side plates of Al and Ag, followed by selective etching of the Al.^[18]^ However, a thickness of 3 nm still corresponds to about 12 layers of Ag atoms.

Moreover, there is a contentious issue whether “ideal” 2D metals can exist at all. Anderson and Mott showed that any disorder or strong Coulomb interactions between electrons could cause a 2D metal to become an insulator. From this perspective any approach towards a 2D metal should aim at minimizing disorder as well as repulsive interactions between electrons, in particular the conduction electrons.[^19^, ^20^, ^21^]

In this context, the topological insulators (TIs) could be of utmost importance.[^22^, ^23^] These materials are envisioned for applications in spintronics or quantum computing due to their topological non‐trivial physical properties.[^24^, ^25^] A TI is a bulk semiconductor with symmetry‐protected metallic surface states. Electrons in these states are immune to backscattering due to a specific symmetry (parity inversion) of the electronic bands at the Fermi level. In a 2D TI or a stack of non‐interacting 2D TIs, which is called a weak 3D TI, the metallic surface states are restricted to the layer edges and helical. Moreover, the spin of the conduction electron is locked orthogonal to its propagation direction. These surface states may possibly be used to transmit (quantum) information without any dissipation on the nanoscale. The stabilization of the electronic surface states can arise for example from spin‐orbit coupling (SOC) generated by the hybridization of the bulk states on both sides of the band gap. If SOC is strong enough, TIs are highly tolerant against defects and far more important are operational at room temperature.[^22^, ^23^]

Currently, high‐quality thin layers of TIs, which are desirable for real industrial applications, are made for example by atomic force microscopy,^[26]^ electrochemistry,^[27]^ but most typically by molecular beam epitaxy (MBE).[^28^, ^29^] In addition, bottom‐up coating approaches including magnetron sputtering,^[30]^ chemical vapor deposition (CVD),[^31^, ^32^] and physical vapor deposition (PVD)^[33]^ are used to develop thin layers based on 1 to 10 μm thick films. Among them, MBE provides the best control of the product quality, yet is very expensive and time‐consuming, and thus cannot meet industrial demands such as low prices, highly efficient production, and effortless implementation. So instead of these mentioned bottom‐up techniques, a top‐down synthesis could be more attractive. Quantitative delamination of a cube of 1 cm^3^ into 10 nm thick platelets will theoretically provide 100 m^2^ of 2D material. Moreover, not all of the relevant materials can be deposited from the gas phase. Possible starting materials are weak 3D TIs that comprise layered structures with strong in‐plane bonds and weak interactions between layers.

**Figure 1:**
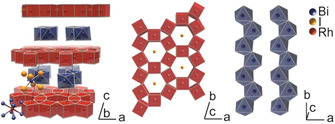
Crystal structure of Bi_14_Rh_3_I_9_ (left) consisting of intermetallic layers ${}_{\infty }{}^{2}[$ (Bi_4_Rh)_3_I]^2+^ (middle) and anionic chains ${}_{\infty }{}^{2}[$ Bi_2_I_8_]^2−^ (right).^[34]^

In 2016, Rasche et al. presented the weak 3D‐TI Bi_14_Rh_3_I_9_, which has a wide topological band gap of about 210 meV.^[34]^ In its crystal structure, intermetallic layers ${}_{\infty }{}^{2}[$ (Bi_4_Rh)_3_I]^2+^ alternate with layers formed by iodobismuthate(III) chains ${}_{\infty }{}^{1}[$ Bi_2_I_8_]^2−^ (Figure 1). The intermetallic layer is three atoms thick and has the same symmetry as graphene (plane group *p*6/*mmm*).^[35]^ The rhodium atom centers a cube of bismuth atoms, and the cubes share common edges to form a planar honeycomb net that hosts an iodide ion in each of its hexagonal voids. It has been demonstrated that this intermetallic layer holds the quantum effect, that is, is a 2D TI.[^34^, ^36^, ^37^, ^38^] There are several aspects that suggest Bi_14_Rh_3_I_9_ as a suitable starting material for a liquid‐phase exfoliation. Quantum‐chemical calculations had shown that strong covalent Rh−Bi bonds together with weaker Bi−Bi bonds stabilize the intermetallic honeycomb net chemically as well as mechanically. The interaction with the anionic spacer is weaker and predominantly electrostatic.[^34^, ^36^, ^37^, ^38^] In addition, the intermetallic layers are tolerant to moderate changes in their electron count, as the existence of various subhalides demonstrates.[^38^, ^39^, ^40^] Further experimental evidence for the chemical stability of the intermetallic honeycomb net has been obtained from previous experiments with the related compound Bi_13_Pt_3_I_7_.^[39]^ In a heterogeneous redox reaction with highly concentrated *n*‐butyllithium (*n*BuLi) in *n*‐hexane, Bi_13_Pt_3_I_7_ was transformed into Bi_12_Pt_3_I_5_. In the topochemical crystal‐to‐crystal transformation the ${}_{\infty }{}^{2}[$ (Bi_4_Pt)_3_I] honeycomb changed its charge from +3 to +4 but remained fully intact, despite the harsh conditions, the considerable volume change, and the mass transport. Mechanical and chemical robustness of the layer as well as its ability to tolerate moderate bending are essential prerequisites for a delamination and exfoliation process. A major issue, however, is the high layer charge, which must be compensated. Since the most top‐down exfoliation methods are based on mechanical treatments and elaborated for layered materials that only bear van der Waals interactions, we developed two new exfoliation routes for layered salts such as Bi_14_Rh_3_I_9_.

## Results and Discussion

### Reductive approach

Since Bi_14_Rh_3_I_9_ is composed of charged layers and chains, there is an unconditional need for a charge compensation during the exfoliation. One option to consider is a reduction reaction that diminishes or eliminates the positive charge of the ${}_{\infty }{}^{2}[$ (Bi_4_Rh)_3_I]^2+^ TI layers, thereby weakening the interaction between the layers. Either anionic ligands with good donor properties or surfactants may be used to stabilize the partially or uncharged intermetallic layers in order to avoid re‐aggregation. These charge‐neutral products may then be cleaved by mechanical activation like sonication, analogue to earlier reported methods used for neutral layered materials to produce freestanding, that is, not substrate‐supported, flakes. However, it is important to note that reduction to an uncharged ${}_{\infty }{}^{2}[$ (Bi_4_Rh)_3_I]^±0^ layer involves the uptake of two electrons per formula unit. This change of the electron count shifts the Fermi level to another, fortunately also non‐trivial band gap of about 100 meV.^[36]^

The first approach represents a heterogeneous reduction process based on the published topochemical approach applied on Bi_13_Pt_3_I_7_.^[39]^ In first orienting experiments, carefully selected Bi_14_Rh_3_I_9_ crystals were suspended in *n*‐hexane before the reducing agent *n*BuLi was injected under inert conditions. During that reductive treatment, the crystallinity and morphology of the starting material changed dramatically. The measured powder X‐ray diffraction (PXRD) pattern could no longer be assigned to the theoretical diffraction pattern of Bi_14_Rh_3_I_9_, but to *rt*‐Bi_2_Rh, *rt*‐Bi_3_Rh, and BiI_3_ (Supporting Information 1). Apparently, Bi_14_Rh_3_I_9_ decomposed.

**Figure 2:**
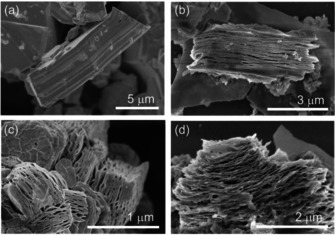
SEM images of (a) untreated Bi_14_Rh_3_I_9_ crystals and (b, c) crystals upon the reductive treatment with *n*‐butyllithium in *n*‐hexane at a maximum of 60 °C. (d) In some areas, thin layer packages were separated.

More promising results were achieved when Bi_14_Rh_3_I_9_ crystals were reacted in *n*‐hexane and *n*BuLi together with a surfactant like poly(vinylpyrrolidone) (PVP). A tendency towards a separation of the dense parent crystal and a puff pastry like morphology were observed (Figure 2).

**Figure 3:**
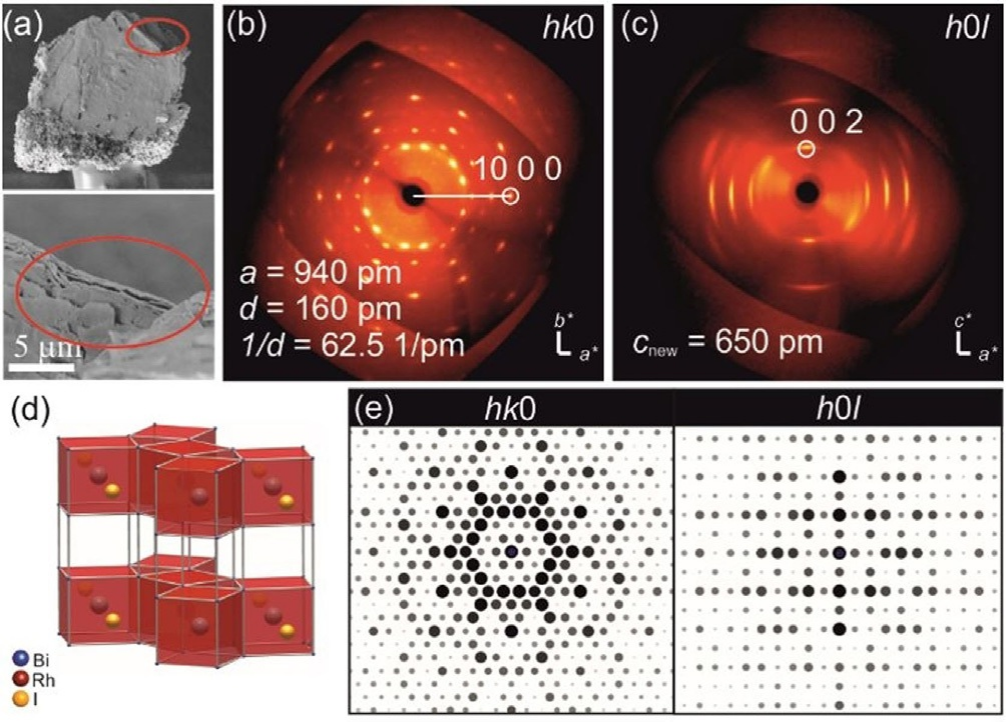
(a) SEM image of the selected, swollen crystal used for SCXRD. (b) XRD pattern of the *hk*0 plane revealing the typical hexagonal pattern of the intermetallic layer. (c) XRD pattern of the *h*0*l* plane showing a decreased c parameter if compared to Bi_14_Rh_3_I_9_. (d) Anticipated crystal structure of Bi_12_Rh_3_I, which is a primitive stacking of intermetallic layers, used for (e) the simulation of the XRD pattern to compare with the experimental data.

In some areas, the flakes were thin enough to see transparent regions when using an acceleration voltage of 2 kV for SEM analyses. Single‐crystal X‐ray and electron diffraction were performed on selected swollen crystals (Figure 3). The collected data indicate the persistence of the intermetallic nets as the typical hexagonal diffraction pattern of the intermetallic layer can still be found in the *hk*0 diffraction plane. However, a dramatic shrinking of the *c* axis to about 650 pm is observed in the *h*0*l* plane, indicating the complete removal of the spacer layers. The chemical composition of the product is either Bi_4_Rh (in a new, probably metastable modification) or Bi_12_Rh_3_I. Local energy‐dispersive X‐ray spectroscopy (EDX) measurements indicated a ratio of Bi/Rh/I=77(3):20(3):3(2) at % (calculated for Bi_12_Rh_3_I: 75:19:6 at %). The mass transport and volume change led to porosity of the solid product and a shift of the remaining intermetallic layers into a primitive stacking sequence. The observed intensities and positions of the reflections in the electron diffraction experiment match very well the simulated electron diffraction pattern for an eclipsed layer stack (Figure 3).

**Figure 4:**
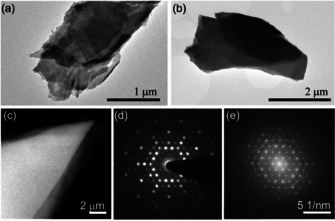
(a, b) Bright‐field TEM image of thin flakes obtained by the reductive approach followed by ultracentrifugation. (c) Dark‐field TEM image of a thin flake showing overlapping areas at the edge. (d) Selected area diffraction pattern revealing the typical hexagonal motif of the intermetallic layer. (e) The inserted Fourier transform of the dark‐field image is depicted.

Eventually, the exfoliation process was boosted by applying mechanical activation provided by sonication. The heated dispersion, that is, swollen crystals in the stabilized liquid, was treated with tip sonication for three hours leading to a total impact energy of about 150 kJ. Indeed, this mechanical exposure was found to be beneficial for the separation of the layered material. The longer the processing time, the higher was the yield of thin layer packages. However, the lateral dimension of the crystalline flakes suffered as a consequence. The flakes did not maintain the size of the parent crystals but showed lateral sizes of about 1–5 μm (Figure 4). The product was highly polydisperse and showed a wide range of flake thicknesses. Thus, the suspension was exposed to ultra‐centrifugation to separate the layer packages with respect to their thickness. Moreover, chemical residues, for example, surfactants in solution, were removed as well. Thin flakes with an estimated thickness of about 50 nm were investigated with transmission electron microscopy (TEM) and selected area electron diffraction (SAED). The diffraction pattern (Figure 4) as well as local EDX analysis on selected flakes (Bi/Rh/I=76(4):18(4):6(3) at %) correspond to those of the porous crystals and show that no further chemical degradation took place upon the mechanical energy inducement.

**Scheme 1:**
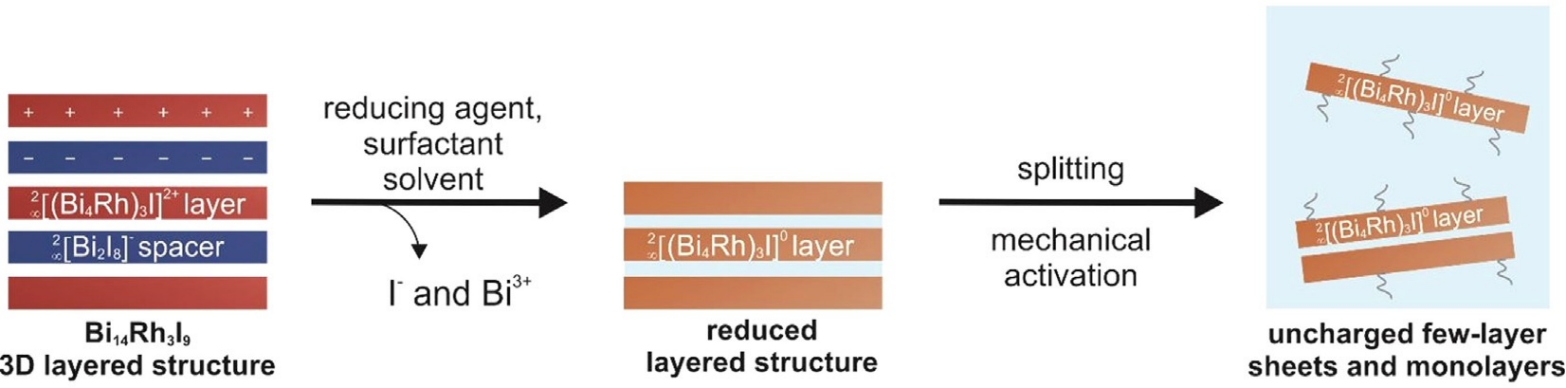
Scheme of the reductive approach to exfoliate Bi_14_Rh_3_I_9_. For charge compensation, electrons are added to the bonding system of the intermetallic layer. The reaction can be amended by surfactants to prevent re‐agglomeration.

In summary, the reductive approach starting from Bi_14_Rh_3_I_9_ crystals led to thin flakes with the nominal composition Bi_12_Rh_3_I (Scheme 1). This is a new compound, which we were not able to synthesize by conventional high‐temperature methods up to now. It represents the spacer‐free stack of intermetallic layers in their reduced form (no layer charge). Details of the crystal structure as well as the electronic and topological properties of Bi_12_Rh_3_I still have to be investigated. The rather violent reaction and the sonomechanical exfoliation again demonstrate the chemical and mechanical robustness of the intermetallic layers.

### Substitutive approach

The second approach describes a substitution of the anionic spacer^[41]^ by organic ligands with charged functional groups, whereby the distance between two adjacent intermetallic layers is increased depending on the size of the ligands. In the case of zwitterions, the negatively charged functional groups of the zwitterion shall saturate the positively charged intermetallic layers. Generally, zwitterions prefer one of their two species in solution depending on the pH‐value. This means that, for instance, a pH‐value change could trigger a splitting of the layered structure, as by way of explanation equally charged functional groups will keep distance due to repulsion. Thus, contrary to the reductive approach the charge is purposefully exploited as a defined predetermined breaking point to exfoliate.

In the first step of the substitutive approach, we aimed at substituting the anionic spacer of Bi_14_Rh_3_I_9_ with betaine (*N*,*N*,*N*‐trimethylglycine). Although the betaine molecule has a positively and a negatively charged end, it is not a classical zwitterion, because it possesses no hydrogen atom, which can migrate and enable isomerization to a form without charge separation.^[42]^ Nonetheless, betaine appears to be small enough to intercalate in Bi_14_Rh_3_I_9_, but large enough to promote splitting by an additional solvent or even separation of the layered compound. Moreover, the anionic carboxyl group can be protonated and thereby the betaine molecule will be charged. After testing several solvents and reaction parameters, the most promising results were observed when treating Bi_14_Rh_3_I_9_ with betaine in dimethylformamide (DMF) in the temperature range between 55 and 60 °C for at least 24 h.

**Figure 5:**
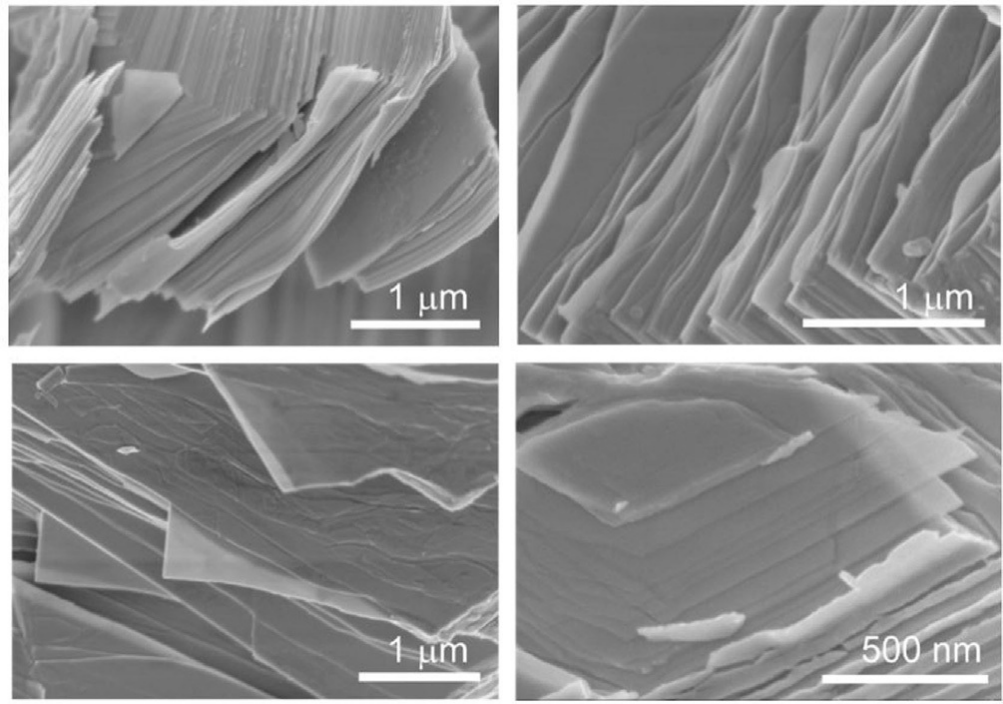
SEM images of Bi_14_Rh_3_I_9_ crystals treated with betaine in dimethylformamide at a temperature of about 55 to 60 °C for at least 24 h revealing the subsequent shifting and splitting of the crystals into different flake thicknesses and lateral dimensions.

The morphology of the Bi_14_Rh_3_I_9_ crystals did not change as dramatically as in the reductive approach. The crystals swelled along the stacking direction and fanned out like a stack of cards, with each card (i.e., layer package) straight and well separated from its neighbors (Figure 5). Individual flakes appeared even almost transparent in the electron beam at an acceleration voltage of about 2 kV.

A higher reaction temperature fostered the expansion of the layered compound, at least to the point at which decomposition and collapse of the expanded stack took place. A higher concentration of betaine gave a higher yield of exfoliated thin layer packages, but only up to the solubility limit of betaine in DMF. The maximum of crystal splitting and the highest yield of separated thin flakes was obtained at 55 °C after 72 h. In experiments in which Bi_14_Rh_3_I_9_ was only heated in DMF, no or only very weak splitting was observed (Supporting Information 2). The same applied to a reaction in which Bi_14_Rh_3_I_9_ was heated in betaine together with another solvent, for example, ethanol or acetone (Supporting Information 3).

**Figure 6:**
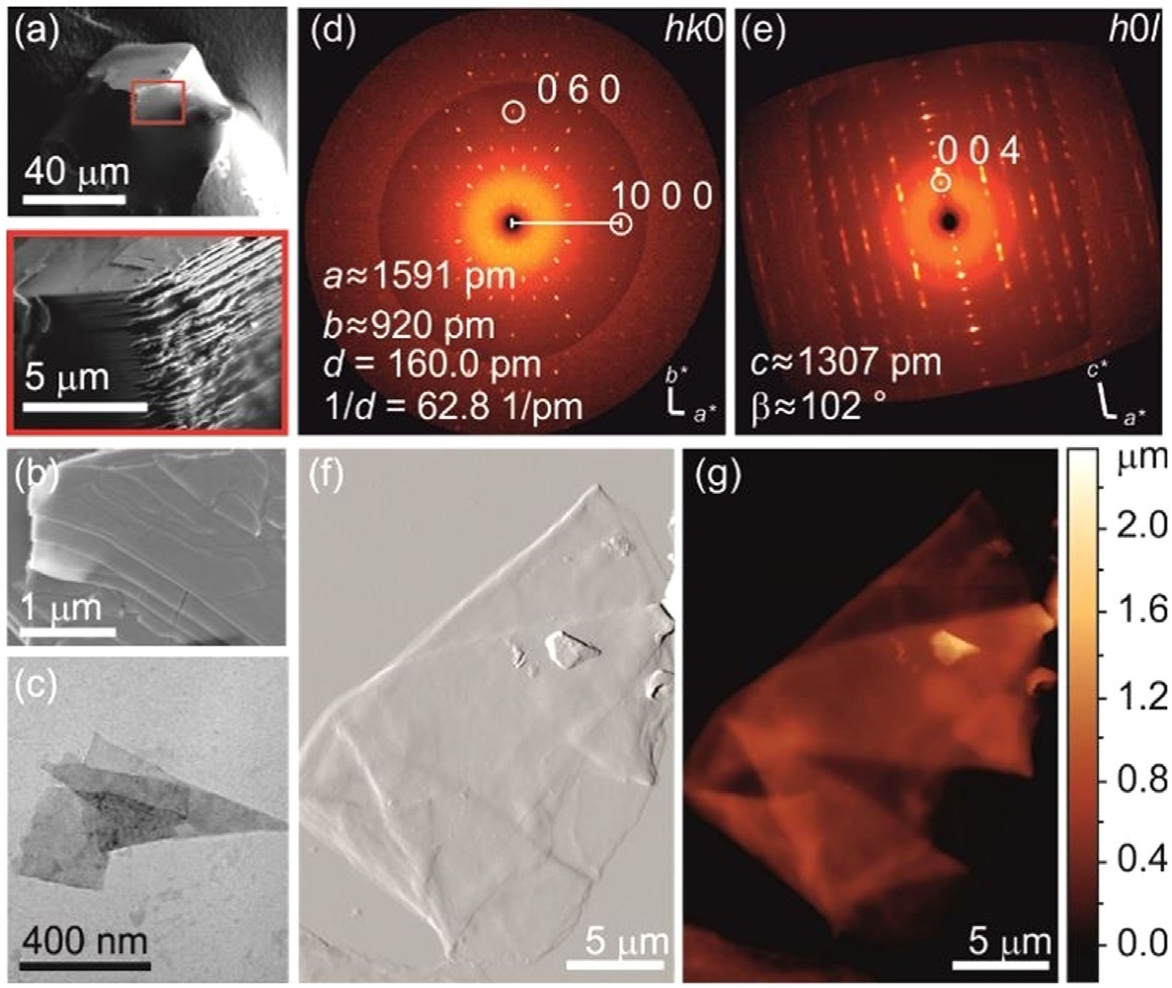
(a–c) SEM and TEM image of swollen crystals and thin flakes resulted from the reaction of Bi_14_Rh_3_I_9_ with betaine in DMF for the SCXRD and AFM measurements. (d, e) Electron diffraction pattern of *hk*0 and *h*0*l* plane that fits well to the unit cell of Bi_14_Rh_3_I_9_. (f, g) AFM data collected on selected thin flakes revealing pleated regions with different thicknesses.

Thus, it can be noted that both organic components are essential for the exfoliation of Bi_14_Rh_3_I_9_, though the mechanism is not known up to now. UV/Vis spectra hint at the formation of an intermediate upon heat treatment of the mixture (Supporting Information 4). Commercially available DMF absorbs at 210 and 244 nm, an aqueous solution of betaine at 210 nm. In contrast, the spectrum of a solution of betaine in DMF that was heated to 60 °C in analogy to the exfoliation conditions shows two additional signals at 252 and 265 nm. PXRD (Supporting Information 5) and EDX analysis confirmed that the chemical composition of the flakes (Bi/Rh/I=55(2):13(3):31(3) at %) agrees to the chemical composition of the starting material Bi_14_Rh_3_I_9_ (54:12:34 at %). The preservation of the entire crystal structure was supported by SCXRD (Figure 6). Apparently, Bi_14_Rh_3_I_9_ intercalated the organic molecules not in every spacer layer, so larger packages of the original structure were maintained. Atomic force microscopy (AFM) studies on selected flakes (no sonication) showed a thickness between 200 and 800 nm and a lateral dimension up to several ten microns (Figure 6). These measurements were hampered by the poor adhesion between the substrate and the produced layer packages, which is a common issue.^[43]^

**Figure 7:**
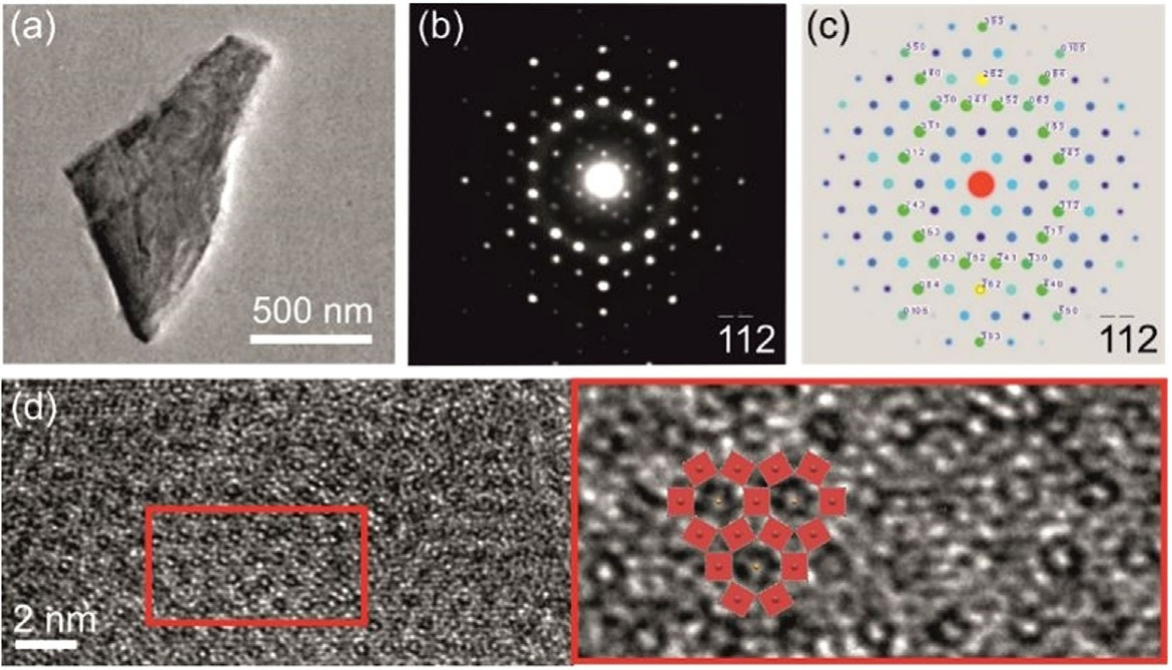
(a) Bright‐field and TEM image of resulting flakes upon the reaction of Bi_14_Rh_3_I_9_ with betaine in DMF followed by a short sonication treatment. (b) The electron diffraction pattern perpendicular to the flake show the typical pseudo‐hexagonal motif of the intermetallic layer of Bi_14_Rh_3_I_9_ in the $\bar{1}\bar{1}$ 2 zone axis orientation proving that the intermetallic net is stable against chemical and mechanical alterations. Weak reflections at low angles from other zones indicate the layer stacking of bulk Bi_14_Rh_3_I_9_. (c) Simulated diffraction pattern of the $\bar{1}\bar{1}$ 2 axis orientation to compare with the experimental diffraction pattern. (d) HRTEM images show bright spots and grey regions building up a hexagonal motif.

To enhance swelling and layer separation, a tip sonication treatment was performed (*t=*30 min, *E=*27 kJ). Subsequently, the dispersion was separated in particle size dependent fractions by (ultra)‐centrifugation. The few‐layer sheets were characterized via TEM analyses (Figure 7). The typical pseudo‐hexagonal electron diffraction pattern in the *hk*0 zone proved that the intermetallic layer still persisted after the sonication treatment. Additional weak reflections at low diffraction angles, which belong to neighboring zones, proved that the layer stacking of bulk Bi_14_Rh_3_I_9_ was preserved. Moreover, high‐resolution (HR)TEM images of distinct areas show an ordered sequence of a hexagonal motif, which fits well with the intermetallic layers of Bi_14_Rh_3_I_9_. EDX analyses of flakes revealed a composition of Bi/Rh/I=66(6):7(2):28(4) at %. These findings strongly suggest that we succeeded in exfoliating thin flakes of the weak 3D topological insulator Bi_14_Rh_3_I_9_.

**Figure 8:**
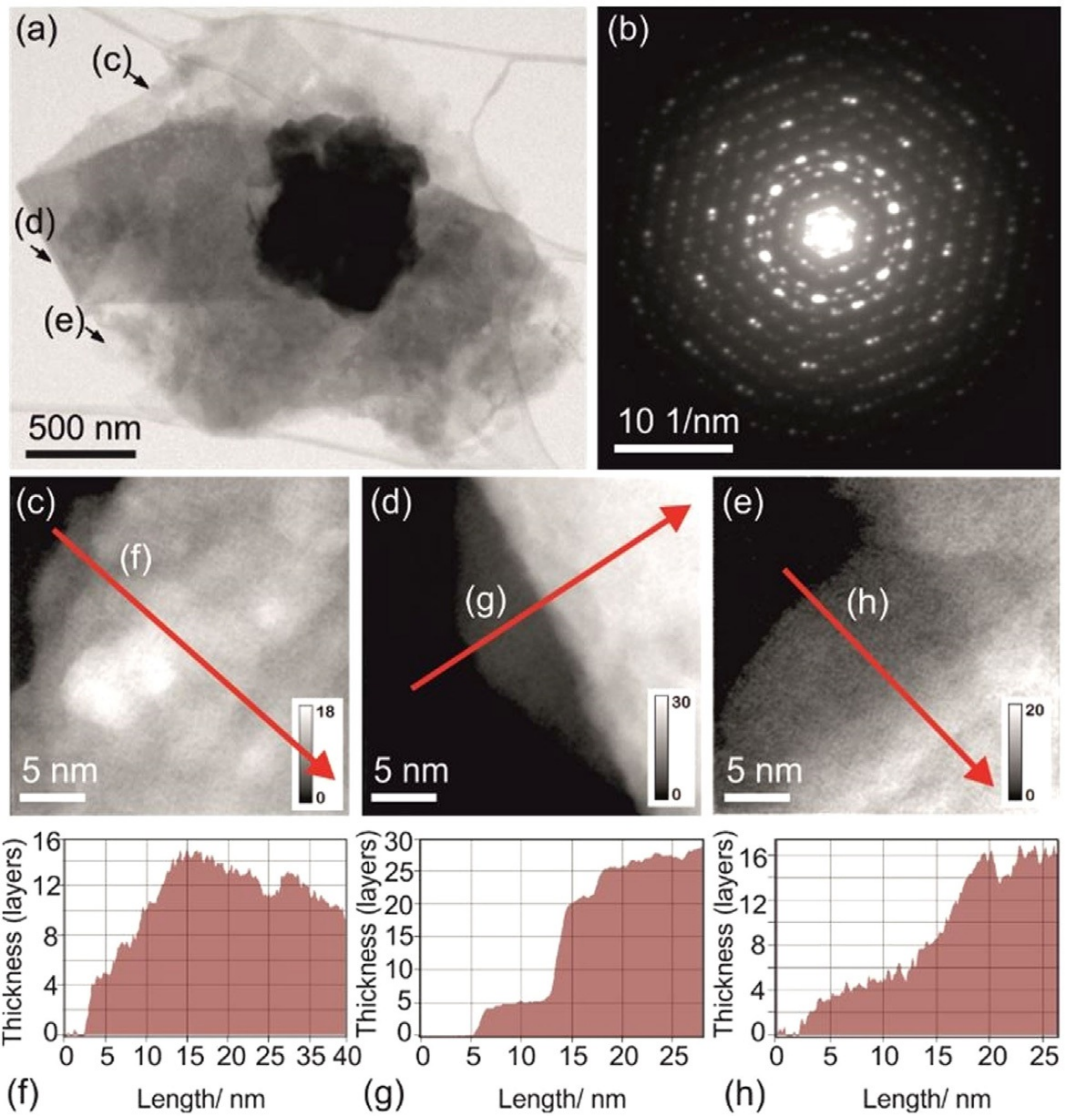
Thickness mapping of the resulting flakes upon the reaction of Bi_14_Rh_3_I_9_ with betaine in DMF followed by a short sonication treatment by off‐axis electron holography. (a) Bright‐field TEM image showing the layered morphology of a typical flake. (b) Electron diffraction pattern of the flake taken in [$\bar{1}\bar{1}$ 2] zone axis orientation of the triclinic crystal structure. (c–e) Line scans. (f–h) Thickness maps in units of layer numbers along line scans indicated by red arrows in (c–e) partially showing thickness plateaus according to a discrete layer number. They were determined by off‐axis EH assuming a mean inner potential of 16.3 V and a layer thickness of 1.25 nm.^[34]^

The thickness of the flakes was determined by off‐axis electron holography (EH) studies.[^44^, ^45^] The thickness profiles (Figure 8) show plateaus at 5, 15, 20, and 30 nm, which correspond to unit cell or TI layer counts of 4, 12, 16, and 24. Such thin flakes have, however, only been found with lateral dimensions up to 200 nm.

**Scheme 2:**
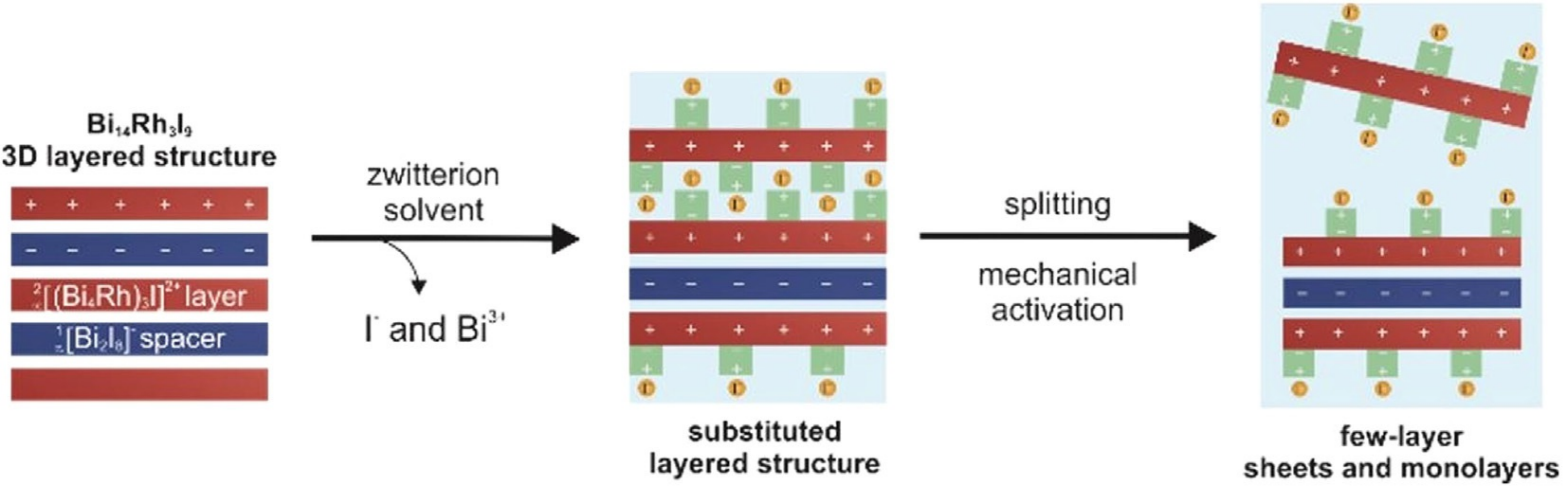
Scheme of the substitutive approach to exfoliate Bi_14_Rh_3_I_9_. Splitting can be amended by suitable solvents and sonomechanical treatments.

In summary, the substitutive approach using a mixture of betaine and DMF promotes splitting of Bi_14_Rh_3_I_9_ crystals (Scheme 2). Subsequent sonication leads to the exfoliation of thin flakes of Bi_14_Rh_3_I_9_ with thickness down to few unit cells. An increase of the lateral dimension requires comprehensive optimization, starting with the growth of the initial Bi_14_Rh_3_I_9_ crystals, then of the exfoliation procedure, and finally of the handling of the few‐layer thin freestanding films.

## Conclusions

Over the last decade, thin films of the strong 3D topological insulators (TIs) Bi_2_Se_3_ and Bi_2_Te_3_ had been grown on substrates through chemical or physical vapor deposition or molecular beam epitaxy (MBE).[^28^, ^29^, ^46^, ^47^] Their structures, which consist of uncharged layers with strong covalent bonds inside and weak van der Waals interactions between them, allowed exfoliation by lithium intercalation^[48]^ or electrochemistry.^[27]^ We now developed two reproducible chemical routes to exfoliate for the first time a weak 3D TI, namely Bi_14_Rh_3_I_9_, into freestanding few‐layer films. Its structure consists of highly charged layers with strong electrostatic interactions between them. Since the methods allow to exfoliate layered salts, they can also be transferred to other materials beyond van der Waals crystals. For both methods, the 2D TI layer of Bi_14_Rh_3_I_9_ is preserved. Hence, we demonstrated the robustness of this intermetallic network regardless of the chemical and sonication treatment. While the reductive approach led to neutral intermetallic layers in the new compound Bi_12_Rh_3_I, the substitutive approach yielded crystalline few‐layer sheets of Bi_14_Rh_3_I_9_. The substitutive method has also considerable advantages regarding the toxicity of the involved chemicals and the difficulty of the preparation. Besides further optimization of the here described liquid‐phase exfoliation process, the appropriate handling of such thin flakes and their implementation into devices has to be developed. In the specific case of a TI, further measures will be necessary to compensate for defects that generate detrimental bulk conductivity.

## Experimental Section

All starting materials and products were handled in an argon‐filled glovebox (MBraun; *p*(O_2_)/*p*_0_<1 ppm, *p*(H_2_O)/*p*_0_<1 ppm). To purify the materials, bismuth (>99.9 %, Merck) and rhodium (>99.9 %, abcr) were treated with hydrogen flow at 220 and 500 °C, while bismuthtriiodide (>99.9 %, Sigma Aldrich) was sublimed at 200 °C prior to the high‐temperature reaction. The synthesis of Bi_14_Rh_3_I_9_ follows the protocol described in the publication of Rasche et al.^[35]^ PXRD was performed on the product to probe the quality.

**Reductive approach**: In a flame‐dried two‐necked 25 mL flask, 0.014 mmol of Bi_14_Rh_3_I_9_ crystals was introduced into 1.242 mmol of distilled *n*‐hexane (for HPLC, HiPersolv Chromanorm) and 1.371 mmol of *n*‐butyllithium (1.6 mol l^−1^, Sigma Aldrich) was injected subsequently with a syringe under argon. Besides, the reaction was amended with surfactants (same amount as Bi_14_Rh_3_I_9_), for example, poly(vinylpyrrolidone) (PVP, Sigma Aldrich). The suspension was heated at 60 °C for 72 h, optionally followed by a 30 min treatment with ultrasonication (Sonoplus, Bandelin) to increase a separation. A part of the solid residue was washed at least three times with absolute ethanol, while the other part was analyzed directly without any further treatment. Size separation was done by (ultra)‐centrifugation (*t=*10–20 min, *f*_max_=10.000–20.000 min^−1^, Beckman Coulter).

**Substitutive approach**: 0.0023 mmol of crystals of Bi_14_Rh_3_I_9_ and 0.085 mmol of betaine powder (≥98 %, Sigma Aldrich) were covered with 8 mL of dimethylformamide (99.8 %, Acros Organics) in a flame‐dried 25 mL flask under argon. Swollen crystals and separated layer packages of Bi_14_Rh_3_I_9_ were obtained after annealing the suspension at 55 °C for 72 h. Swelling and exfoliation were increased by sonication treatments (*t*_max_=30 min, *E*_max_=30 kJ, Sonopuls, Bandelin). Afterwards, size separation was done by (ultra)‐centrifugation (*t=*10, 20, 40 min, *f*_max_=5.000, 10.000, and 20.000 min^−1^, Beckman Coulter). The washing procedure is similar to the one of the reductive approach, see above.

**Powder X‐ray diffraction**: The PXRD data [CuK_α1_, *λ*=154.0562 pm, *T=*296(1) K] were collected using an X′Pert Pro diffractometer [PANalytical, Bragg–Brentano geometry, Ge(220) hybrid monochromator, fixed divergence slits, PIXcel detector]. The samples were ground and fixed on single‐crystal silicon sample holders. The Powder Diffraction File Database (PDF) and the Inorganic Crystal Structure Database (ICSD) were used for phase identification.

**Single‐crystal X‐ray diffraction**: Single‐crystal X‐ray diffraction measurements [MoK_α_, *λ*=71.073 pm, *T=*296(1) K] were performed on a four‐circle diffractometer Kappa APEX II CCD (Bruker) equipped with a graphite(002) monochromator, and a CCD detector. Single‐crystals were selected and glued on top of a glass fiber, which was then mounted to a goniometer head. Some of the crystals were washed with ethanol or cut in inert oil in order to provide suitable quality and size for the measurement. Polarization and Lorentz factors corrections were performed within the APEX2 program.^[49]^

**Scanning electron microscopy and energy‐dispersive X‐ray spectroscopy**: SEM was performed using an electron microscope SU8020 from Hitachi equipped with multi detector system for secondary and low‐energy backscattered electrons while an Oxford Silicon Drift Detector X‐MaxN was used for the semi‐quantitative EDX. Suspensions washed with ethanol were dropped on a silicon wafer fixed on a carbon pad under argon, which were then settled on an aluminum sample holder. The EDX data were evaluated within the AZtec software package.^[50]^

**Transmission electron microscopy and off‐axis electron holography**: TEM imaging was performed in an FEI Titan^3^ 80–300 (ThermoFisher Company) microscope at 300 kV acceleration voltage. The instrument was equipped with an image aberration corrector allowing for a spatial resolution down to 0.7 Å in the bright field mode. We conducted both high‐resolution TEM imaging and electron diffraction studies to resolve the crystal structure. To record the diffraction patterns we therefore used the three‐lens condenser system to generate a parallel illumination at larger probe diameters of about 400 nm, significantly reducing the beam induced damage. To index the diffraction patterns we employed the SingleCrystal software package (internationally registered trademark of CrystalMaker Software Ltd, UK) allowing for simulating electron diffraction patterns.

Off‐axis electron holography^[45]^ was carried out also at the FEI Titan^3^ 80–300 using an acceleration voltage of 300 kV. To image larger regions of the few 100 nm sized flakes, the holographic field‐of‐view was enhanced by using the so‐called Lorentz mode (lower magnifying Lorentz‐lens switched on, higher magnifying “normal” objective lens switched off). In order to generate an electron hologram, that is, the interference pattern between the object‐exit wave and object‐free reference wave, a bias voltage of 180 V was applied to the electro‐static Moellenstedt biprism. Phase images of the Bi_14_Rh_3_I_9_ object exit waves were then reconstructed from the holograms by Fourier method^[46]^ that provide a resolution of about 5 nm. Exploiting the linear relationship between phase shift *φ* and thickness *t*, according to *φ*=*C*_*ϵ*_⋅*V*_0_⋅*t*, the thickness of the flakes was determined with known interaction constant *C*_*ϵ*_=0.0065 rad V^−1^ nm^−1^ at 300 kV and mean inner potential *V*_0_=16.3 V of Bi_14_Rh_3_I_9_.

**Atomic force microscopy**: AFM measurements were taken with a Dimension Icon (Fa. Bruker) with a cantilever. The images were plotted with the NanoScope 9.3 Software. Therefore, samples were prepared by spin‐coating on poly(dimethylsiloxane) (PDMS) stripes, which were fixed in a provided module. The PDMS surface was shortly heated using a hotplate to evaporate solvent residues.

**UV/Vis spectroscopy**: UV/Vis absorption spectra were measured with a UV‐1650PC spectrometer (Shimadzu) using quartz glass SUPRASIL cuvettes (Hellma Analytics). The measurements were taken in the wavenumber range from 200 nm to 800 nm.

## Conflict of interest

The authors declare no conflict of interest.

## Supporting information

As a service to our authors and readers, this journal provides supporting information supplied by the authors. Such materials are peer reviewed and may be re‐organized for online delivery, but are not copy‐edited or typeset. Technical support issues arising from supporting information (other than missing files) should be addressed to the authors.

Supplementary
