# Supplementary material for: Freestanding Nanolayers of a Wide‐Gap Topological Insulator through Liquid‐Phase Exfoliation
Source: Chemistry. 2020 Dec 4;27(2):794–801. doi: 10.1002/chem.202004320 (PMC7839554; doi:10.1002/chem.202004320)
Supplement: Supplementary file 1 — Supplementary [file CHEM-27-794-s001.pdf]

Chemistry–A European Journal

Supporting Information

Freestanding Nanolayers of a Wide-Gap Topological Insulator through Liquid-Phase Exfoliation

Mai Lê Anh,[a] Pavel Potapov,[b] Daniel Wolf,[b] Axel Lubk,[b, c] Bernhard Glatz,[d] Andreas Fery,[d] Thomas Doert,[a] and Michael Ruck*[a, c, e]

Supporting Information

Reductive Approach

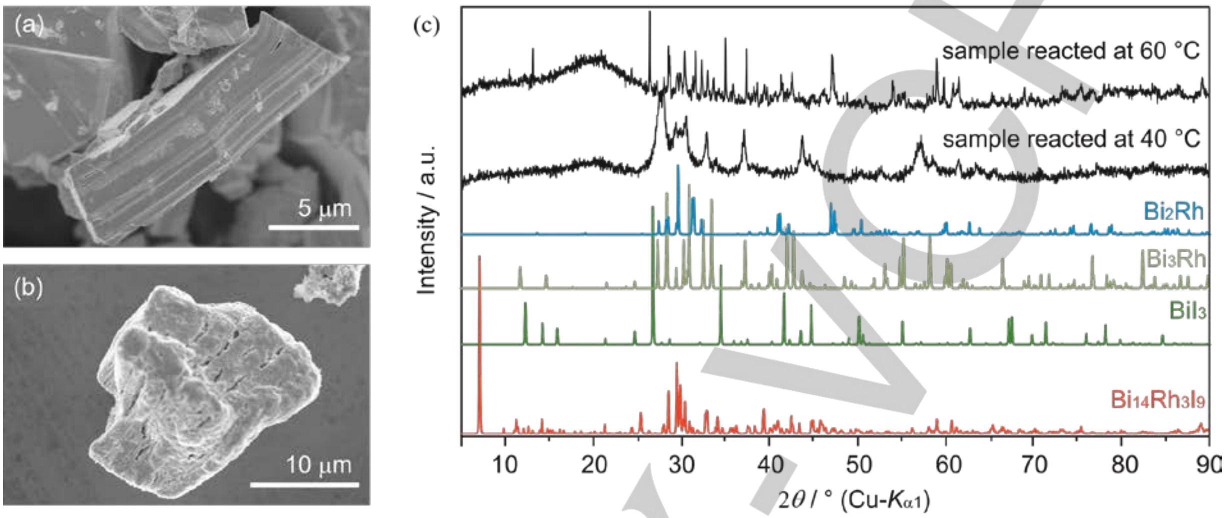

Supporting 1. SEM images of (a) pristine $Bi_{14}Rh_3I_9$ crystals and (b) crystals treated with *n*-BuLi in *n*-hexane. The corresponding PXRD patterns of samples (black coloured) are displayed in comparison to the theoretical pattern of $Bi_{14}Rh_3I_9$ (red coloured), BiI_3 (green coloured), Bi_3Rh (olive coloured) and Bi_2Rh (blue coloured) showing that $Bi_{14}Rh_3I_9$ decomposes upon the reductive treatment.

Substitutive Approach

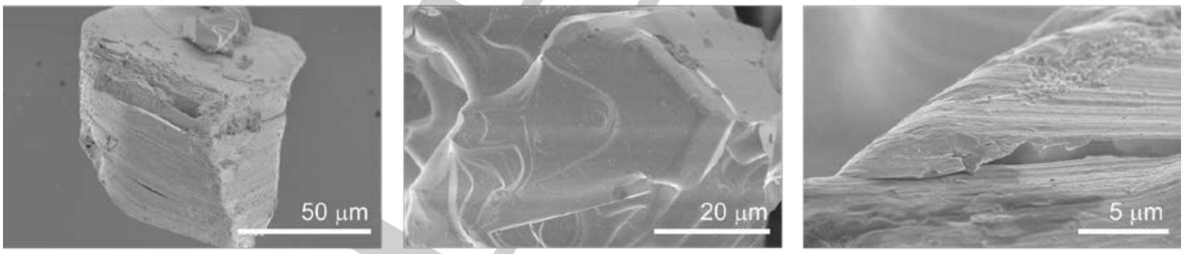

Supporting 2. SEM images of $Bi_{14}Rh_3I_9$ crystals that were heated in neat DMF for several hours to investigate which role DMF plays in this reaction. From this experiments, it can be deduced that DMF is not solely responsible for the splitting behaviour, but can be supportive.

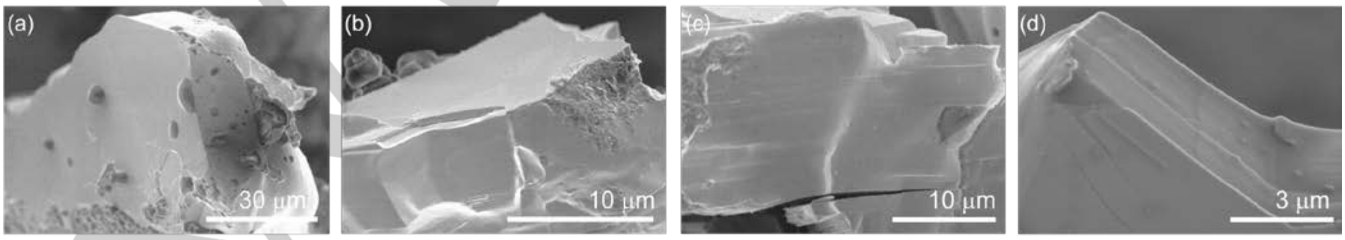

Supporting 3. SEM images of $Bi_{14}Rh_3I_9$ crystals that were reacted with betaine in (a) methanol, (b) ethanol, (c) acetonitrile and (d) dimethyl sulfoxide under the same reaction conditions as used for dimethylformamide showing no shifting or splitting along the crystal's edges.

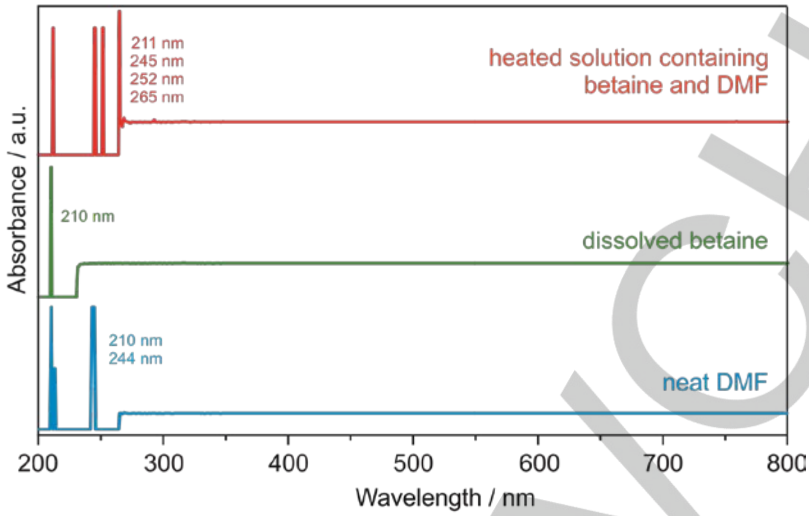

Supporting 4. UV/VIS spectra of neat DMF (blue coloured), betaine dissolved in water (green coloured) and a heated solution containing betaine and DMF (red coloured) indicating a formation of an intermediate since two additional signals were detected.

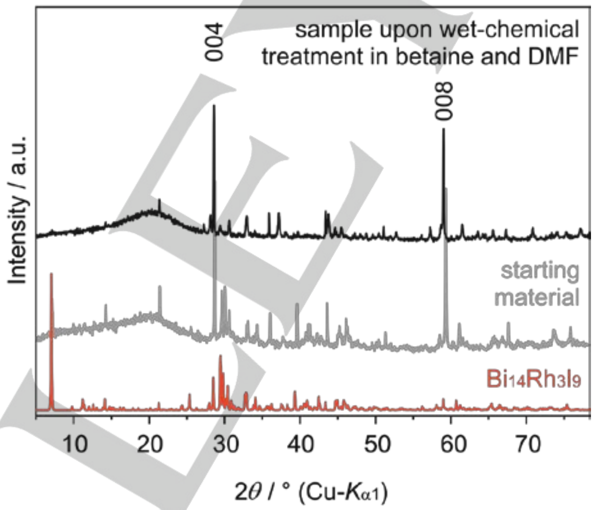

Supporting 5. PXRD pattern of the fanned out crystals shown in Figure 6 compared to the experimental diffraction pattern of the starting material $Bi_{14}Rh_3I_9$ and its theoretical diffraction pattern.

WILEY-VCH
